# A Review of the Current Literature on Pleomorphic Adenoma

**DOI:** 10.7759/cureus.42311

**Published:** 2023-07-22

**Authors:** Dheer S Kalwaniya, Reena Meena, Devender Kumar, Aditya Tolat, Satya V Arya

**Affiliations:** 1 General Surgery, Vardhman Mahavir Medical College and Safdarjung Hospital, New Delhi, IND

**Keywords:** parotidectomy, facial nerve, mixed tumor, salivary gland, pleomorphic adenoma

## Abstract

Pleomorphic adenomas (PA) are the most common benign salivary gland tumors. They arise from the major salivary glands, as well as the minor salivary glands. They may arise rarely from the palate, oral cavity, neck, and nasal cavity also. Yet, the fourth, fifth, and sixth decades of life are the most common for them to manifest. Forty percent of them occur in males and 60% in females. It is a benign mixed tumor, which has three components: an epithelial component, a myoepithelial cell component, and a mesenchymal component. A fibrous capsule separates these cells from the surrounding tissues. It generally presents as a slowly progressing painless swelling, which is well-delineated, nonsymptomatic, and not involving the facial nerve. Salivary gland tumors can be accurately diagnosed before surgery using fine-needle aspiration (FNA), ultrasonography (USG), and computed tomography (CT) scan. Calponin, cluster of differentiation 9 (CD9), glial fibrillary acidic protein (GFAP), Mcl-2, metastasis suppressor gene (NM23), p63, S-100, smooth muscle actin (SMA), and SRY-box transcription factor 10 (SOX10) exhibit the majority of the positive reactions in pleomorphic adenomas. The diagnostic marker pleomorphic adenoma gene 1 (PLAG1) is frequently employed since it is specific for pleomorphic adenoma. Although benign, these epithelial tumors have a propensity to recur and undergo malignant transformation if incompletely excised, leading to increased morbidity in these patients. A review of the consensus guidelines and literature was conducted, and the online literature on the subject from 2002 was included. This article is not a complete review of all the available literature; rather, it is a comprehensive review of the topic.

## Introduction and background

Pleomorphic adenomas (PA) are the most common benign salivary gland tumors [1]. They arise from the major salivary glands, but they may also arise from the minor salivary glands [2]. It is mostly situated in the parotid glands (85%), followed by the minor salivary glands (10%) and the submandibular glands (5%) [3].

Pleomorphic adenoma was first termed by Willis [4]. Its name comes from the architectural pleomorphism that may be seen with a light microscope [5]. Myoepithelial and epithelial cells combine to form this benign mixed tumor. These cells are isolated from the surrounding tissues by a fibrous capsule [6].

The hard palate is the most common site for mixed tumor in the small salivary glands. Another region that is frequently affected by the tumor is the lips; a small minority of tumors are located in the oral cavity, neck, and nasal cavity [5,7,8]. Other intraoral sites also include the buccal mucosa, tongue, floor of the mouth, tonsil, pharynx, retromolar area, and gingival and nasal cavities [5,7-9].

Most tumors originate in the superficial lobe, but more rarely, these tumors may involve the deep lobe of the parotid gland, and they may grow medially and involve the parapharyngeal space [10]. Pleomorphic adenoma often manifests as a swelling that grows slowly, is asymptomatic, and does not affect the facial nerve [11].

It is a single cell, rather than the simultaneous multiplication of cancerous epithelial and myoepithelial cells, that differentiates into either an epithelial or a myoepithelial cell [12].

It was previously known by other names such as mixed tumor, enclavoma, branchioma, endothelioma, and enchondroma [13].

The tumor is made up of three different cell types: epithelial, myoepithelial, and mesenchymal. Conceptually, PA recognition is based on the recognition of these three elements. The epithelium in the loose fibrous stroma of the myxoid, chondroid, or mucoid types exhibits various patterns in the histological presentation of PA. Myoepithelial cells have a polygonal shape and a light eosinophilic cytoplasm. Microscopic identification is required for a definitive diagnosis of a pleomorphic adenoma [14].

Pleomorphic adenomas may occur at any age, but mainly, they affect patients in the fourth, fifth, and sixth decades of life. Forty percent of them occur in males and 60% in females. Pleomorphic adenoma tumors clinically are painless, well-delineated, and covered with normal mucous membrane. Ulcerations can occasionally be seen. Single and movable related nodules are present. Unlike minor gland tumors, major gland tumors are typically encapsulated [15].

The only effective course of treatment in these situations is the complete excision of the lesion. To limit recurrence and the development of cancer, one should endeavor to prevent any breaks in the continuity of the lesion and completely remove it [6].

Although benign, these epithelial tumors have a propensity to recur and undergo malignant transformation if incompletely excised, leading to increased morbidity in these patients [16].

The purpose of this study is to determine the prevalence of pleomorphic adenoma in extraparotid sites, gender, the age distribution, the most common complaints, treatment options, the best treatment to prevent recurrence, benign to malignant transformation, the immunohistochemistry of pleomorphic adenoma, and the role of radiation therapy.

## Review

Material and methods

In order to find the cases of pleomorphic adenoma, a search of the English-language literature was conducted using the PubMed database. Also, the terms mixed tumor and salivary gland tumors were used as synonyms. PA, mixed tumor, reviews, and case reports were the search phrases utilized to find relevant literature. All the reviewed articles were from 2002 onward.

Inclusion criteria include all pleomorphic adenoma case reports, literature review of articles on pleomorphic adenoma, English-language reports or literature, and at least six-month follow-up after surgical treatment.

Exclusion criteria include all the papers that were written in languages other than English, pleomorphic adenoma-related dissertations or textbook pages, and pleomorphic adenoma articles before 2002.

Result

In the Journal of Web Semantics, our search came up with 130 papers, of which 70 references were kept for abstract reading and just 46 full papers were reviewed, which fulfills all the criteria for inclusion. From the obtained information, Table 1 and Table 2 were created. Table 1 contained information about extraparotid pleomorphic adenoma, which includes the following data: author, year of publication, age, gender, lesion associated with pain, location, encapsulation, invasion into adjacent tissues, and treatment chosen. Table 2 contained information about parotid pleomorphic adenoma.

**Table 1:** The list of the articles reviewed for extraparotid pleomorphic adenomas F, female; M, male

Investigator	Pain	Site	Age years	Gender	Encapsulated	Invasion into adjacent tissue	Treatment	Recurrence
Aryal et al., 2023 [17]	No	Nasal septum	69	F	Yes	No	Wide local excision with a clear margin	No
Berrerhdoche et al., 2022 [18]	No	Soft palate	26	F	Yes	No	Wide surgical excision	No
Salahuddin and Sairin, 2022 [19]	No	Nasal septum	22	F	Yes	No	Wide surgical excision	No
Ghadirimoghadam et al., 2022 [20]	No	Hard palate	27	F	Yes	No	Surgical excision and enucleation	No
Sigdel et al., 2022 [21]	No	Nasal septum	72	F	No	No	Endonasal surgical excision	No
Ito et al., 2022 [22]	No	Breast	43	F	Yes	No	Surgical excision	No
Lygeros et al., 2021 [23]	No	Maxillary sinus	60	M	Yes	No	Surgical excision	No
Dongol and Karki, 2021 [24]	No	External auditory canal	23	M	Yes	No	Surgical excision	No
Kubota et al., 2021 [25]	No	Nasal septum	49	F	Yes	No	Surgical excision with cartilage resection	No
Gelidan and Arab, 2021 [26]	No	Upper lip	46	M	Yes	No	Surgical excision	No
Vijayakumar et al., 2022 [27]	No	Uvula	43	M	Yes	No	Surgical excision	No
Angula et al., 2020 [28]	No	Left retroauricular	53	F	Yes	No	Extended left parotidectomy	No
Júnior et al., 2020 [29]	No	Left buccal mucosa	50	F	Yes	No	Surgical excision	No
Khanal, 2019 [30]	No	Right submandibular gland	38	M	Yes	No	Surgical excision	No
Mouzali et al., 2019 [31]		Right ala nasi	20	M	Yes	No	Surgical excision	No
Zhu et al., 2018 [32]	No	Right main bronchus	38	F	Yes	No	Surgical excision	No
Taiwo et al., 2018 [33]	No	Right upper lip	33	M	Yes	No	Surgical excision	No
Ali et al., 2016 [34]	No	Right lower lobe and the lung	60	M	Yes	No	Surgical excision	No
Jain et al., 2015 [35]	No	Left side of the face	50	F	Yes	No	Surgical excision	No
Verma et al., 2014 [36]	No	Left side of the cheek	42	F	Yes	No	Surgical excision	No
Saito et al., 2014 [37]	No	External auditory canal	40	M	Yes	No	Retroauricular surgical approach	No
Sunil and Gopakumar, 2013 [38]	No	Angle of the right mandible	62	F	Yes	No	Surgical excision	No

**Table 2:** List of articles reviewed for parotid pleomorphic adenomas FNAC: fine-needle aspiration cytology

Author	Title	Conclusion
Speight and Barrett, 2020 [39]	Salivary gland tumours: diagnostic challenges and an update on the latest WHO classification	Size of tumor a strong predictor of prognosis; rule of 4 cm
Sungur et al., 2002 [40]	Clinicopathological evaluation of parotid gland tumors: a retrospective study	After benign tumors, adenocystic carcinoma (55.7%) was a common malignant tumor; no significant difference in complication rate between the anterograde and retrograde approaches in facial nerve injury
Stathopoulos et al., 2018 [41]	Partial superficial, superficial, and total parotidectomy in the management of benign parotid gland tumors	Transient facial nerve more significant after total and superficial parotidectomy than partial superficial parotidectomy; surgical outcome better with superficial parotidectomy
Sharma et al., 2011 [42]	An objective assessment of proximal and distal facial nerve exploration during superficial parotidectomy	Out of 39 patients, 29 patients underwent conventional proximal nerve exploration; both approaches consumed on the same operative time, while blood loss was lesser in the distal approach. No significant difference in surgical margin status was noticed between the two techniques (P>0.05)
Kalwaniya et al., 2020 [43]	Factors associated with facial nerve palsy in patients undergoing superficial parotidectomy for pleiomorphic adenoma: our experience of eight and half years	Tumor size was >4 cm, tumor depth was >2 cm, and reoperation had higher chances of immediate facial nerve paresis or paralysis
Meyer et al., 2021 [44]	2021 update on diagnostic markers and translocation in salivary gland tumors	-
Shashinder et al., 2009 [45]	A review of parotid tumours and their management: a ten-year-experience	FNAC diagnostic in 90% of the patients with sensitivity and specificity of 76% and 96%, respectively; temporary facial palsy was found in 39% of the patients, while 4% of the patients had permanent facial palsy; 2.6% rate of recurrence of pleomorphic adenoma
Foresta et al., 2014 [46]	Pleomorphic adenoma and benign parotid tumors: extracapsular dissection vs superficial parotidectomy--review of literature and meta-analysis	Superficial parotidectomy had a higher rate of recurrence and a higher incidence of cranial nerve 7 paralysis, and Frey syndrome is more common after superficial parotidectomy

The conclusion drawn from the case reports mentioned in Table 1 was that the mean age of the development of pleomorphic adenoma was 43.90 years (range: 20-72). The male-to-female ratio of pleomorphic adenoma was 9:13, clearly indicating a female preference. The nasal septum was the most often reported extraparotid site, followed by the palate, upper lip, right submandibular region, external auditory canal, maxillary sinus, bronchus, breast, and uvula. The patient's primary complaint did not involve pain. The risk of recurrence was reduced by complete tumor excision with a clear margin.

Review

Epidemiology

The annual incidence of salivary gland tumors, an uncommon neoplasm of the head and neck, ranges from 7.03 to 8.58 per 100,000 [47]. These tumors have a high degree of morphological variation, multiple similar characteristics, and low incidence rates [39].

Males were shown to have a higher frequency of parotid tumors (both benign and malignant diseases). With mean ages of 35 years (22-50 years) and 51 years (28-68 years), respectively, benign tumors were frequently reported in middle-aged and elderly patients, while malignant tumors were more frequently noted in elder patients. There is no dominant left or right side of involvement in either malignant or benign tumors [40].

Accessory salivary gland tumors are quite uncommon. The palate is the preferred location for accessory salivary tumors, with more than 50% of the accessory glands evenly distributed across the hard and soft palates (in two-thirds of the cases). Between the third and fifth decades of life is the average time frame for the occurrence of pleomorphic adenoma of the soft palate or roof of the mouth, which affects females slightly more than males [48,49].

Risk Factors

Risk factors might be inherited or environmental. One well-known environmental risk factor is exposure to therapeutic radiation [50]. After radiation exposure, the incidence increases from 15 to 20 years, but the precise etiology is still unknown. The disease's etiology is also suspected to involve genetic predisposition, cigarette use, and exposure to toxins [51].

According to studies, people who received radiation therapy for childhood cancers were more likely to acquire salivary gland tumors [50]. Smoking, surprisingly, does not appear to be strongly associated with the growth of pleomorphic adenomas, but it does significantly contribute to the growth of Warthin's tumor, a different kind of benign salivary gland tumor that frequently affects the parotid gland [52].

Although infection with the simian virus 40 (SV40), a highly oncogenic tumor virus, appears to play a role in the development of pleomorphic adenomas, Epstein-Barr virus (EBV) and cytomegalovirus do not appear to be connected with this risk factor. The simian virus 40 (SV40) was originally found in monkeys but accidentally transferred to many humans who received an anti-polio vaccine. Study suggests that even those who did not receive the polio vaccine may have contracted the SV40 [51]. The SV40 tag sequence was frequently identified by polymerase chain reaction (PCR) in pleomorphic adenomas but not in normal salivary gland tissue, indicating that the simian virus 40 likely contributes to the development or progression of pleomorphic adenomas [51]. Pleomorphic adenomas exhibit a familial component, according to the research, and their two routes of inheritance are somatic mutation and autosomal dominant inheritance [53]. If pleomorphic adenomas are inherited autosomally, a patient's offspring would have a 50% chance of inheriting the condition [54].

Clinical Features

The primary site of occurrence is in the parotid gland, where it manifests as a swelling on the ramus of the mandible in front of the ear and occurs in the superficial lobe. A hard, uneven nodular lesion is how it presents itself. If cystic degeneration is shallow and does not exhibit fixation, it can be palpated. In most cases, there are no symptoms, including no pain or facial nerve involvement [55].

In the study by Sungur et al., an asymptomatic parotid mass was the primary complaint in 77.3% of the cases. Thirty-two patients (22.7%) showed signs of edema, fluctuation, soreness, and/or pain. Four cases (1.7%) of facial paralysis were noted. Intraoral involvement was seen in three (1.3%) of the patients with deep lobe tumors. The symptoms could appear at any age between a few weeks and 12 years [40].

Diagnosis

The diagnosis is made both on tissue sampling and radiographic studies.

Imaging

Because of their rarity and morphological diversity, they are challenging to diagnose. Imaging plays a significant part in the staging process of salivary gland cancer and offers essential information for the proper localization of salivary gland tumors (e.g., localization in superficial and deep lobes) and distinguishing between benignancy and malignancy.

Pleomorphic adenomas seen on ultrasound generally have a hypoechoic texture. They typically have a well-defined border with lobules, either with or without posterior acoustic enhancement [56].

In a computed tomography (CT) scan, a pleomorphic adenoma typically shows up as a globular mass with smoothly marginated or lobulated homogeneous soft tissue density. Larger masses can exhibit necrosis. There are not many common calcification foci. While larger tumors exhibit less pronounced and delayed enhancement, smaller tumors have early homogeneous significant enhancement [56].

The method of choice for analyzing salivary gland cancers is magnetic resonance imaging (MRI). MRI is similar to CT; smaller masses appear well-circumscribed and homogeneous, whereas larger tumors appear heterogeneous [56].

Regular pre-contrast MRI is crucial for determining the precise location of salivary gland cancers, their locoregional extent, and tumor margins. Ito et al. reported a very rare case report in 2022 of a pleomorphic adenoma in the breast; similar to other tumors, an accurate preoperative diagnosis was challenging. The pathology and MRI features of a pleomorphic adenoma of the breast may resemble those of other breast tumors, including phyllodes tumor, complicated primary sarcoma, encapsulated papillary carcinoma, mucinous carcinoma, intracystic papilloma, and fibroadenoma. To more precisely distinguish benign pleomorphic adenoma of the breast from malignant tumors in this instance, a study of the MRI data, including the dynamic contrast enhancement pattern, may be necessary [22].

In cases of malignant transformation, post-contrast short tau inversion recovery (STIR) images aid in defining the perineural dissemination of the tumor [56].

For differentiating recurrence from post-treatment changes, MRI is helpful. The assessment of skull-base cortical intrusions often involves post-contrast computed tomography (CT) imaging; they show diffuse mottling radiolucency and cupped-out bone resorption. PET-CT is an efficient way to identify distant metastases of cancer of the salivary gland [57].

Fine-Needle Aspiration Cytology (FNAC)

Techniques such as fine-needle aspiration (FNA) and core needle biopsy are used for obtaining a tissue sample that can be carried out in an outpatient clinic These procedures have relatively low rates of tumor seeding [58,59].

Fine-needle aspiration cytology (FNAC) is the primary diagnostic tool for parotid gland lesions [60]. Its value is increased when reported by a cytopathologist with experience in the diagnosis of salivary gland disease. Because of the possibility of cellular spreading and subsequent problems, several experts do not recommend incisional biopsy [61-63]. The preferred method for acquiring a histology sample and evaluating the subtype and severity of the tumor is therefore fine-needle aspiration (FNA) [61,62].

Salivary gland tumors can be accurately diagnosed before surgery using fine-needle aspiration (FNA). In order to make therapeutic decisions, it aids in separating benign from malignant tumors [64]. Moreover, FNA helps to limit the necessity for surgical intervention, which can lower treatment expenses [65].

With FNA, it is possible to identify the tumor's malignancy with a sensitivity of about 90%. The histological type of the tumor is more precisely determined by a core needle biopsy, which is more invasive but has a diagnosis accuracy of about 97% [56].

Sandhu et al. classified the cases of pleomorphic adenoma for cellularity as mild, moderate, and marked and for the ratio of the epithelium to the mesenchymal component. Pleomorphic adenoma displayed a variety of morphological alterations, including squamous metaplasia (17.9%), mucinous metaplasia (2.9%), oncocytic change (5.9%), hyaline globule (2.9%), large cells (4.47%), cystic degeneration (22.38%), and plasmacytoid cell differentiation (32.83%) [66].

Immunohistochemical Pattern

PA reacts mostly positively to calponin, cluster of differentiation 9 (CD9), glial fibrillary acidic protein (GFAP), Mcl-2, metastasis suppressor gene (NM23), p63, S-100, smooth muscle actin (SMA), and SRY-box transcription factor 10 (SOX10). Pleomorphic adenoma gene 1 (PLAG1) is specific for PA and is therefore widely used as a diagnostic marker. Amylase; discovered On GIST1, encodes the chloride channel protein anoctamin 1 (DOG1); high-mobility group AT-hook 2 (HMGA2); receptor tyrosine kinase (KIT); and myeloblastosis viral oncogene homolog (MYB) are sometimes positive. The usually negatively reacting markers are carbonic anhydrase VI, LPLUNC1, short-palate lung nasal epithelial clone 1 (SPLUNC1), and SPLUNC2 [44].

Benign to Malignant Transformation

Salivary gland pleomorphic adenoma (SGPA), the most common salivary gland tumor, has a notoriously high probability of developing into carcinoma ex-pleomorphic adenoma (CEPA), the fifth most common salivary gland carcinoma. Rapid growth in the presence of a known PA and/or the new onset of pain, facial nerve paralysis, skin ulceration or fixation, or lymphadenopathy is additional manifestations that may take place. Moreover, the patients may have a history of PA procedures in the past. Just 3% of SGPAs recur at 12.5-year follow-up, and of those, 6% appear to exhibit malignant change, making this risk of transformation rare. The recognition of a pleomorphic adenoma component in malignant salivary gland tumors, which are subsequently classified as "CEPA," is the main support for the concept that SGPA undergoes malignant transformation. The benign pleomorphic adenoma component of these tumors changes morphologically and molecularly to the malignant carcinoma component [67,68].

According to Valstar et al., the chronological genetic steps of the advancement of SGPA to a malignant salivary gland tumor, as demonstrated for the first time in the paper, are also linked to the incidence of *TP53* mutations. The *LIFR/PLAG1* gene fusion, *PIK3R1* mutation, and allelic imbalance pattern shared by all lesions indicated that they all originated from the main SGPA. It is tempting to hypothesize that the proliferation was driven by the combination of the *LIFR/PLAG1* gene fusion and the *PIK3R1* mutation with the loss of the other allele, while the additional *TP53* mutations started the malignant transformation [67].

Majority of the time, PLAG1 or high-mobility group AT-hook 2 (HMGA2) translocations are known to be obscured by pleomorphic adenomas. Such tumors carry a high risk of morbidity and frequently recur if they are not promptly treated with wide safety margins. These tumors have a high mortality rate and are an aggressive subtype of malignant salivary gland tumors [68].

A marker called mucin 1 (MUC1)/DF3 may be used to identify pleomorphic adenomas that have undergone potential recurrence or malignant transformation [54].

*Treatment* 

The most preferred treatment for the mass is surgical excision with adequate margins of tumor-free tissue. The surgeon should keep the tumor's size and position in mind, as well as its vascularity, malignancy, and relationship to important structures including the oropharyngeal airway, neck, and vascular bundle, while deciding on the best surgical approach [20]. Previously, the standard procedure for treating benign parotid gland tumors was to remove the tumor entirely while leaving the capsule in place, which had a very high recurrence rate. It is not the preferred course of treatment [69].

A recent meta-analysis has shown that as long as the tumor is less than 4 cm in diameter and does not affect the facial nerve, extracapsular dissection may be a good alternative for treating unilateral benign parotid tumors of the superficial lobe [69]. Consequently, it is acceptable to infer that a more involved technique than extracapsular dissection is required for tumors larger than 4 cm in order to prevent long-term harm to one or more branches of the facial nerve.

In cases where PA affects the superficial lobe of the parotid gland, a superficial parotidectomy with facial nerve preservation is carried out. It not only reduces the duration of surgery but also reduces the risk of damaging the facial nerve as fewer branches are dissected [70].

In their prospective study, Stathopoulos et al. confirmed that transient nerve palsy is lower after partial superficial parotidectomy than after total and superficial parotidectomy [41]. It is well known that the risk of injury to the nerve is also proportional to the length of nerve dissected [71].

Bittar et al. came to the conclusion in their study that facial nerve damage is significantly more likely to occur in tumors that are 3.0 cm in length and/or 2.0 cm in depth. After a superficial parotidectomy, there is a substantially increased risk of facial palsy developing from secondary surgery for recurrent malignancies [72]. While another study done by Kalwaniya et al. concluded that the patients with tumors that were >4 cm in size and >2 cm in depth or who had undergone resection had an increased risk of experiencing facial nerve paresis or paralysis immediately. Of the patients, 11.3% experienced facial nerve paresis or paralysis right after parotidectomy [43].

According to a meta-analysis published in 2002, extracapsular dissection is not advised for tumors that are tightly adherent to the nerve since it does not allow for complete removal, leading to disruptions in the capsule and pseudopodia [73].

Although the surgeon intends to remove the tumor with a cuff of parotid tissue during a partial superficial parotidectomy, exposure and tumor rupture are still a possibility and an unwanted outcome. Over a 20-year follow-up, there is an 8% chance of recurrence after a rupture [74]. If malignancies affect the deep lobe, a total parotidectomy is performed; they may grow medially and involve the parapharyngeal space. As the pleomorphic adenoma can be present in the small salivary glands, submandibular glands, plates, nasal septum and is rare in the external auditory tube and lacrimal gland, the treatment for the smaller salivary gland pleomorphic adenomas involves extensive local excision along with the affected bone or periosteum. PA has a good prognosis with a 95% cure rate [67].

Radiotherapy's function is still debatable [75]. Jackson et al. administered postoperative radiation in the patients whose histology revealed that the specimen's resection margins were not clear of tumor or in those who had tumor spilling during surgery [75].

According to Douglas et al., when total extirpation is not achievable, when the sacrifice of the facial nerve is required, or after multiple recurrences, irradiation is advised [76].

Histopathology

The excised mass was sent for histopathological confirmation. The mass appears in the cut section as an irregular, ovoid mass with well-defined edges. It might or might not be entirely enclosed. It may have a rubbery, meaty, or mucoid consistency, with spots of hemorrhage and infarction scattered throughout [55]. Typically, the epithelium forms sheets or ductal structures with an epithelial layer of myoepithelial cells surrounding an inner layer of large, cuboidal cells. Often, eosinophilic secretory material is seen in the ducts. The myoepithelial cells might be transparent, hyaline, spindle-shaped, polygonal, or other shapes. The tumor may occasionally be predominately composed of hyalinized, myxoid/mucoid, or cartilaginous mesenchymal cells. The myxoid, chondroid, and hyalinized stromata are believed to be produced by myoepithelial cells [34].

Complications

In a retrospective analysis done by Shashinder et al., the most prevalent post-parotidectomy problem was partial facial nerve palsy, which was observed in 27 patients (35%), followed by recurrence (8%), and complete facial palsy in 4% of the cases. Several issues were observed, including a hemorrhage in one patient and four individuals with wound infections [45].

Another study by Sharma et al. on 39 patients revealed that one patient had Frey syndrome, while four and one patient, respectively, had temporary and permanent facial nerve function disrupted. After surgery, sialocele developed in two individuals [42].

Follow-Up

Long-term follow-up is necessary to look for any signs of local recurrence after treatment because recurrence rates increase with time [77]. According to certain research, imaging (a CT scan or an MRI) was repeated during the follow-up [78].

Recurrence

The risk of recurrence was correlated with tumor site, age at diagnosis, and margin status [67]. Pleomorphic adenoma simple enucleation is linked to high recurrence rates, between 8% and 45%, which are decreased to less than 5% with superficial parotidectomy and even lower to 0.4% with total parotidectomy [79].

Due to the low event rate (0.15%), risk factors for the malignant transformation of recurrent pleomorphic adenoma could not be identified [67].

## Conclusions

Pleomorphic adenoma of the small salivary gland is an extremely rare benign tumor, which is more common in females. In Pennsylvania, a hereditary predisposition is also evident. Unlike other benign tumors, it has the peculiarity of recurring and converting into malignancy. Therefore, wide surgical excision and long-term follow-up are crucial for proper patient management. The patient should undergo a superficial or total parotidectomy to lower the risk of recurrence. Patients who are more likely to experience a recurrence due to positive margins, tumor spillage during surgery, or incomplete resection may benefit from radiation. The early identification of benign to malignant conversion can be made by conducting long-term follow-up, which will enable the patient to receive treatment sooner.
